# Solid Fuel Use and the Progression of Multimorbidity in Middle-Aged Chinese Participants: A Prospective Cohort Study

**DOI:** 10.3389/ijph.2022.1605206

**Published:** 2023-01-09

**Authors:** Tingting Wu, Yue Zhang, Yaguan Zhou, Zifan Zhang, Yangyang Cheng, Xiangtong Liu, Xiaolin Xu

**Affiliations:** ^1^ School of Public Health The Second Affiliated Hospital, Zhejiang University School of Medicine, Hangzhou, Zhejiang, China; ^2^ School of Public Health, Capital Medicine University, Beijing, China

**Keywords:** multimorbidity, cohort study, Chinese, household air pollution, solid fuel use

## Abstract

**Objectives:** This study aimed to examine the association of solid fuel use for cooking and heating with the progression of multimorbidity.

**Methods:** A total of 5,437 participants from the China Health and Retirement Longitudinal Study were included. Multivariate logistic regression models were used to estimate the odds ratios (ORs) and 95% confidence intervals (CIs) for the associations of the independent and joint effects of solid fuel use for cooking and heating with the progression of multimorbidity.

**Results:** The proportion of participants reporting solid fuel use for both cooking and heating was 59.0% at baseline. Solid fuel use for both cooking and heating was associated with the progression of multimorbidity (adjusted OR: 1.42, 95% CI: 1.19–1.70), compared with clean fuel use for both.

**Conclusion:** Solid fuel use for cooking and heating play an important role in the progression of multimorbidity. Therefore, solid fuel reduction should be considered in developing multimorbidity control and prevention programmes.

## Introduction

Using solid fuels (e.g., coal, wood, charcoal, dung, and crop residues [1]) for cooking or heating could lead to household air pollution, which is one of the most important risk factors for the rising number of chronic diseases. This is because solid fuels can produce an array of pollutants (like PM2.5, black carbon, and carbon monoxide), particularly in inadequately ventilated homes [2]. According to China’s National Energy Administration, China has provided full electricity access to all residents in December 2015 [3]. However, approximately 361 million people in China continued to use solid fuel for cooking and heating, leading to 8.74 million disability-adjusted life-years (DALYs) and .36 million deaths in 2019 [2]. Solid fuel for cooking has been a major public health problem in China and other developing countries [4, 5], and burning solid fuel for heating in winter or rainy seasons is also common in China. For example, burning solid fuels in heating stoves or “kangs” in the winter is still the most common home-heating practice [6, 7]. Previous studies have explored the health impacts of solid fuel use, but most of them did not distinguish between the effect of using solid fuel for cooking and for heating [8–11]. The independent and joint effects of solid fuel use for cooking and heating on health remain largely unknown.

Evidence has emerged that solid fuel use was associated with single chronic conditions, such as hypertension, diabetes, lung disease, liver disease, heart disease, arthritis, and asthma [12–15]. With the aging of the population, the co-existence of two or more chronic conditions in the elderly, defined as multimorbidity, has become more common worldwide, with the prevalence of multimorbidity in China ranging from 57% to 81% [16, 17]. Several studies showed that multimorbidity led to reduced quality of life such as poor functional status, increasing cost of health services such as longer hospital stays, and higher levels of primary care than single conditions [16, 18–20]. This challenged the current public health system, which paid more attention to single conditions than multimorbidity.

This study aimed to examine the independent and joint effects of solid fuel use for cooking and heating on the progression of multimorbidity using the China Health and Retirement Longitudinal Study (CHARLS), a nationally representative prospective study in China.

## Methods

### Study Population

This longitudinal cohort study was conducted based on the harmonized data (2011–2018) from CHARLS developed by the Gateway to Global Aging Data. Detailed information on CHARLS has been described elsewhere [21]. Briefly, the CHARLS enrolled participants from 450 urban communities and rural areas in 28 provinces of China. Information on health status, socio-demographic characteristics, and lifestyle factors were collected. The baseline survey with 17,708 individuals was conducted between June 2011 to March 2012 and the follow-up surveys were carried out every 2 years (2013, 2015, and 2018).

**FIGURE 1:**
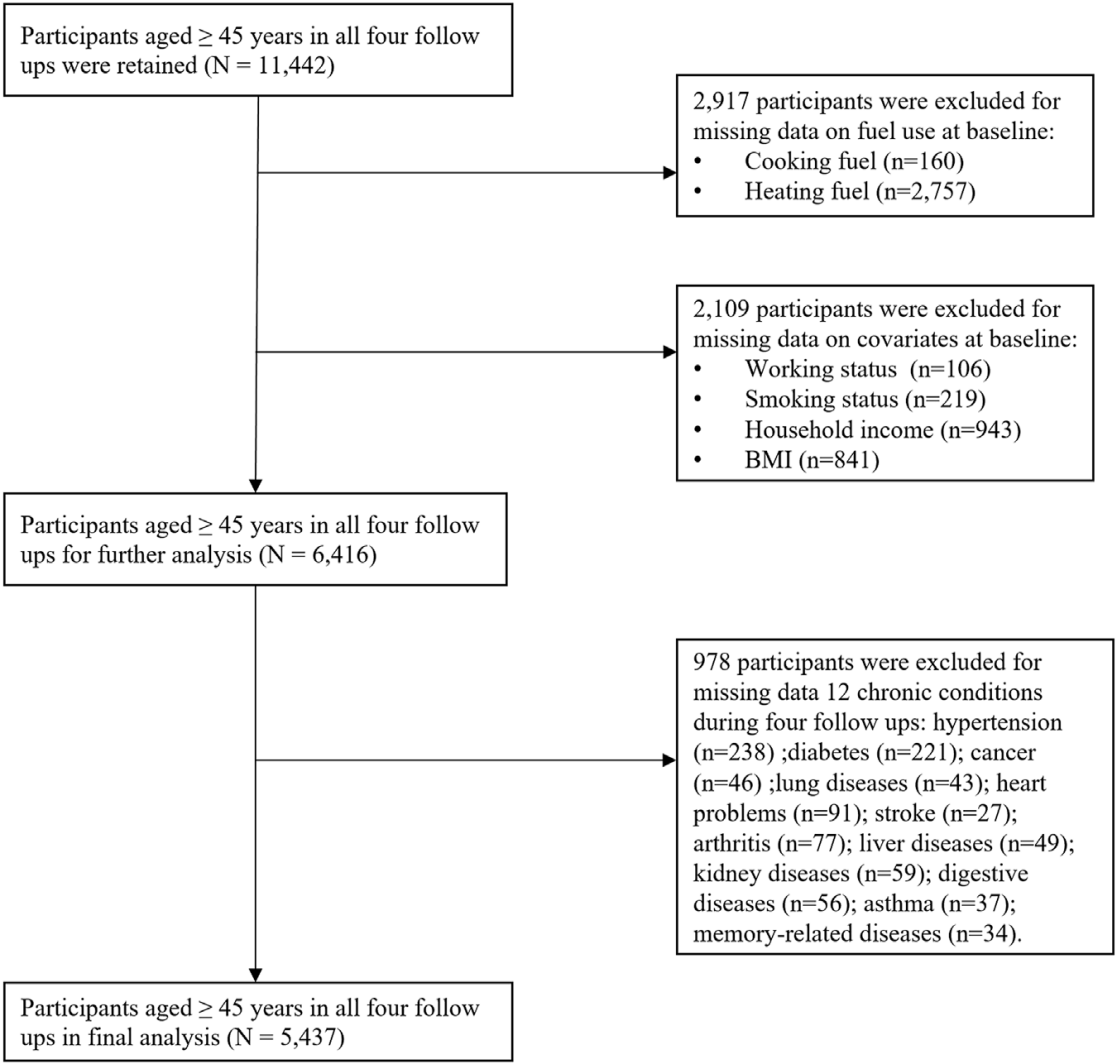
Flowchart of the participants included in the present study (China Health and Retirement Longitudinal Study, China, 2011–2018).

A total of 11,442 participants aged 45 years and above who responded to the four surveys were included in our study. The exclusion criteria were: 1) individuals with missing values on solid fuel use; 2) individuals with missing values on covariates; 3) individuals with missing values on information about chronic conditions. Finally, a total of 5,437 participants were included in the analysis (Figure 1).

### Assessment of Solid Fuel Use

Information on solid fuel use was collected through a questionnaire assessment of CHARLS, in which the fuel for cooking was collected across four waves (2011, 2013, 2015, and 2018), and fuel for heating was collected in 2011 and 2015. Using the following questions: “what is the main source of cooking fuel?” and “what is the main heating energy source?”. Based on previous studies [11, 22], we categorized cooking fuel as either solid fuel (crop residue/wood; or coal) or clean fuel (natural gas; marsh gas [CH4]; liquefied petroleum gas; or electric); heating fuel was also categorized as either solid fuel (crop residue/wood; or coal) or clean fuel (solar; natural gas; liquefied petroleum gas; or electric) [23]. The joint effect of solid fuel use for cooking and heating was categorized as both clean fuel use, either solid fuel use and both solid fuel use. In the current study, the baseline survey of fuel use was used for the main analysis.

### Assessment of Multimorbidity Progression

Based on previous studies about the association of household air pollution with chronic conditions and the disease burden of household air pollution [12, 24, 25], 12 chronic conditions were selected to define multimorbidity in this study, including hypertension, diabetes or high blood sugar, cancer or malignancies, chronic lung diseases (e.g., chronic bronchitis and emphysema), heart problems (e.g., heart attack, coronary heart disease, angina, congestive heart failure, or other heart problems), stroke, arthritis or rheumatism, liver diseases, kidney diseases, digestive diseases (e.g., stomach or other digestive diseases), asthma, and memory-related disease (e.g., Alzheimer’s disease, Brain atrophy, and Parkinson’s disease). In each wave (2011, 2013, 2015, and 2018) participants were asked whether they were diagnosed with a list of the above 12 chronic conditions. Stable condition progression was defined as not developing any new conditions during follow-up. Single condition progression was defined as developing one condition for participants with no condition at baseline. Multimorbidity progression was defined as developing two or more chronic conditions for participants with no conditions or developing the new condition(s) for participants with one or more conditions at baseline.

### Covariates

Covariates were collected at baseline. According to previous studies [22, 26], covariates include socio-demographic variables (age, sex, education level, marital status, working status, household income, and residence) and lifestyle factors (smoking status, drinking status, and body mass index [BMI]). Sex was reported as male and female. Education level was categorized as less than lower secondary, upper secondary & vocational, and tertiary. Working status was categorized as unemployed/retired/never worked and employed. Household income was categorized by the quartile. Marital status was categorized as married/partnered and others (separated, divorced, widowed, and never married). The residence was categorized as living in urban or rural. Smoking status was categorized as ever/never smoking and current smoking. Drinking status was categorized as never drinking and ever drinking. BMI was classified according to the criteria for Chinese adults [27]: underweight: <18.5 kg/m^2^; normal weight: 18.5–23.9 kg/m^2^; overweight: 24–27.9 kg/m^2^; obese: ≥28 kg/m^2^.

### Statistical Analysis

Baseline characteristics were described as mean with standard deviation (SD) or numbers (percentages) according to solid fuel use for cooking and heating at baseline (2011), multimorbidity progression during follow-up (2011–2018), and four waves (2011, 2013, 2015, and 2018), respectively. Furthermore, the baseline characteristics were also presented according to the sample excluding or not excluding missing data. Differences among the groups were compared using the Student’s t-test or Analysis of Variance for continuous variables and the Chi-squared test for categorical variables.

Multivariate logistic regression models were used to calculate odds ratios (ORs) with 95% confidence intervals (CIs) for the association of the independent and joint solid fuel use for cooking and heating with the progression of multimorbidity, compared with clean fuel use. Model 1 was unadjusted. Model 2 was adjusted for age, sex, education levels, marital status, working status, household income, and residence. Model 3 was further adjusted for smoking status, drinking status, and BMI. Solid fuels for cooking and heating were mutually adjusted in all models.

To test the robustness of our results, this study further conducted several additional analyses with the fully adjusted model: 1) analyzing the joint effect of solid fuel use for cooking and heating on the incidence of 12 single chronic conditions; 2) analyzing the independent effect of each specific type of fuel for cooking and heating on multimorbidity progression; 3) subgroup analyses by age, sex, education levels, marital status, working status, household income, and residence, smoking status, drinking status, and BMI among the joint effect of solid fuel use for cooking and heating on multimorbidity progression; 4) two sensitivity analyses of the joint effect of solid fuel for cooking and heating on multimorbidity progression, one repeated the analysis in participants without the selected conditions at baseline and the other one conducted by defining electric or solar as clean fuel. Additionally, we also explored the association between the duration of solid fuel use for cooking from 2011 to 2018 and multimorbidity progression. The duration of solid fuel use for cooking was categorized as 0, 1-6, and 7 years or more. It was calculated based on the fuel types used for cooking in four waves [28]. For example, if individuals used clean fuel for cooking in all four waves, then the duration of solid fuel use for cooking was 0 years. Likewise, the duration of solid fuel use for cooking was >7 years if individuals used solid fuel for cooking in all four waves.

Statistical analyses were performed using SAS (version 9.4, SAS Institute Inc.). A two-sided result with *p* < .05 in all statistical tests were considered statistically significant.

## Results

### Baseline Characteristics of Study Populations

The baseline characteristics of participants were compared according to the solid fuel use for cooking and heating at baseline (Table 1) and the progression of multimorbidity during follow-up (Table 2), respectively. At baseline, 64.1% of participants reported using solid fuel for cooking, 78.5% reported using solid fuel for heating, and 59.0% reported using solid fuel for both cooking and heating. A total of 3,011 (55.4%) participants experienced multimorbidity progression during follow-up. In general, participants who used solid fuel were more likely to have lower levels of education, live in rural areas, be employed, and have lower household income (Table 1). Participants who experienced multimorbidity progression were more likely to be employed and non-drinkers (Table 2). Additionally, the baseline characteristics of participants were also presented according to the sample, excluding or not excluding missing data (Supplementary Table S1) and four waves (2011, 2013, 2015, and 2018) (Supplementary Table S2).

**TABLE 1:** Baseline characteristics of participants according to solid fuel use for cooking and heating at baseline (China Health and Retirement Longitudinal Study, China, 2011).

Characteristics	Total	Cooking			Heating			Cooking and heating			
		Clean fuel	Solid fuel	*p*-value^a^	Clean fuel	Solid fuel	*p*-value^b^	Both clean fuel use	Either solid fuel use	Both solid fuel use	*p*-value^c^
No. (%)	5,437 (100)	1,953 (35.9)	3,484 (64.1)		1,169 (21.5)	4,268 (78.5)		892 (16.4)	1,338 (24.6)	3,207 (59.0)	
Age (mean ± standard deviation[SD])	57.9 ± 8.5	56.7 ± 8.4	58.6 ± 8.6	<.01^d^	56.8 ± 8.7	58.3 ± 8.5	<.01^d^	56.2 ± 8.4	57.5 ± 8.5	58.6 ± 8.5	<.01^d^
Sex (%)				.25^e^			.40^e^				.47^e^
Male	2,541 (46.7)	933 (47.8)	1,608 (46.2)		559 (47.8)	1,982 (46.4)		433 (48.5)	626 (46.8)	1,482 (46.2)	
Female	2,896 (53.3)	1,020 (52.2)	1,876 (53.8)		610 (52.2)	2,286 (53.6)		459 (51.5)	712 (53.2)	1,725 (53.8)	
Education (%)				<.01^e^			<.01^e^				<.01^e^
Less than lower secondary	4,959 (91.2)	1,692 (86.6)	3,267 (93.8)		1,023 (87.5)	3,936 (92.2)		768 (86.1)	1,179 (88.1)	3,012 (93.9)	
Upper secondary & vocational training	446 (8.2)	243 (12.4)	203 (5.8)		135 (11.5)	311 (7.3)		115 (12.9)	148 (11.1)	183 (5.7)	
Tertiary	32 (.6)	18 (.9)	14 (.4)		11 (.9)	21 (.5)		9 (1.0)	11 (.8)	12 (.4)	
Marital status (%)				.38^e^			.43^e^				.37^e^
Married/partnered	4,930 (90.7)	1,780 (91.1)	3,150 (90.4)		1,067 (91.3)	3,863 (90.5)		811 (90.9)	1,225 (91.6)	2,894 (90.2)	
Others	507 (9.3)	173 (8.9)	334 (9.6)		102 (8.7)	405 (9.5)		81 (9.1)	113 (8.4)	313 (9.8)	
Working status (%)				<.01^e^			<.01^e^				<.01^e^
Unemployed/retired/never worked	1,586 (29.2)	673 (34.5)	913 (26.2)		380 (32.5)	1,206 (28.3)		319 (35.8)	415 (31.0)	852 (26.6)	
Employed	3,851 (70.8)	1,280 (65.5)	2,571 (73.8)		789 (67.5)	3,062 (71.7)		573 (64.2)	923 (69.0)	2,355 (73.4)	
Residence (%)				<.01^e^			<.01^e^				<.01^e^
Urban	1,624 (29.9)	960 (49.2)	664 (19.1)		630 (53.9)	994 (23.3)		537 (60.2)	516 (38.6)	571 (17.8)	
Rural	3,813 (70.1)	993 (50.8)	2,820 (80.9)		539 (46.1)	3,274 (76.7)		355 (39.8)	822 (61.4)	2,636 (82.2)	
Household income (%)				<.01^e^			<.01^e^				<.01^e^
Quartile 1 (lowest)	1,360 (25.0)	286 (14.6)	1,074 (30.8)		132 (11.3)	1,228 (28.8)		83 (9.3)	252 (18.8)	1,025 (32.0)	
Quartile 2	1,359 (25.0)	456 (23.3)	903 (25.9)		218 (18.6)	1,141 (26.7)		150 (16.8)	374 (28.0)	835 (26.0)	
Quartile 3	1,360 (25.0)	538 (27.5)	822 (23.6)		340 (29.1)	1,020 (23.9)		259 (29.0)	360 (26.9)	741 (23.1)	
Quartile 4 (highest)	1,358 (25.0)	673 (34.5)	685 (19.7)		479 (41.0)	879 (20.6)		400 (44.8)	352 (26.3)	606 (18.9)	
Smoking status (%)				.12^e^			.21^e^				.25^e^
Ever/never smoking	3,727 (68.5)	1,364 (69.8)	2,363 (67.8)		819 (70.1)	2,908 (68.1)		629 (70.5)	925 (69.1)	2,173 (67.8)	
Current smoking	1,710 (31.5)	589 (30.2)	1,121 (32.2)		350 (29.9)	1,360 (31.9)		263 (29.5)	413 (30.9)	1,034 (32.2)	
Drinking status (%)				.12^e^			.32^e^				.29^e^
Never drinking	3,624 (66.7)	1,276 (65.3)	2,348 (67.4)		765 (65.4)	2,859 (67.0)		581 (65.1)	879 (65.7)	2,164 (67.5)	
Ever drinking	1,813 (33.3)	677 (34.7)	1,136 (32.6)		404 (34.6)	1,409 (33.0)		311 (34.9)	459 (34.3)	1,043 (32.5)	
BMI (%)				<.01^e^			<.01^e^				<.01^e^
Underweight	358 (6.6)	105 (5.4)	253 (7.3)		54 (4.6)	304 (7.1)		44 (4.9)	71 (5.3)	243 (7.6)	
Normal weight	2,900 (53.3)	962 (49.3)	1,938 (55.6)		585 (50.0)	2,315 (54.2)		437 (49.0)	673 (50.3)	1,790 (55.8)	
Overweight	1,552 (28.5)	631 (32.3)	921 (26.4)		393 (33.6)	1,159 (27.2)		304 (34.1)	416 (31.1)	832 (25.9)	
Obese	627 (11.5)	255 (13.1)	372 (10.7)		137 (11.7)	490 (11.5)		107 (12.0)	178 (13.3)	342 (10.7)	
Hypertension (%)				.38^e^			.50^e^				.22^e^
No	4,124 (75.9)	1,468 (75.2)	2,656 (76.2)		878 (75.1)	3,246 (76.1)		677 (75.9)	992 (74.1)	2,455 (76.6)	
Yes	1,313 (24.1)	485 (24.8)	828 (23.8)		291 (24.9)	1,022 (23.9)		215 (24.1)	346 (25.9)	752 (23.4)	
Diabetes (%)				<.01^e^			.34^e^				.05^e^
No	5,151 (94.7)	1,829 (93.7)	3,322 (95.4)		1,101 (94.2)	4,050 (94.9)		837 (93.8)	1,256 (93.9)	3,058 (95.4)	
Yes	286 (5.3)	124 (6.3)	162 (4.6)		68 (5.8)	218 (5.1)		55 (6.2)	82 (6.1)	149 (4.6)	
Cancer (%)				.12^e^			.54^e^				.08^e^
No	5,402 (99.4)	1,936 (99.1)	3,466 (99.5)		1,160 (99.2)	4,242 (99.4)		886 (99.3)	1,324 (99.0)	3,192 (99.5)	
Yes	35 (.6)	17 (.9)	18 (.5)		9 (.8)	26 (.6)		6 (.7)	14 (1.0)	15 (.5)	
Lung diseases (%)				<.01^e^			.02^e^				<.01^e^
No	4,926 (90.6)	1,807 (92.5)	3,119 (89.5)		1,080 (92.4)	3,846 (90.1)		833 (93.4)	1,221 (91.3)	2,872 (89.6)	
Yes	511 (9.4)	146 (7.5)	365 (10.5)		89 (7.6)	422 (9.9)		59 (6.6)	117 (8.7)	335 (10.4)	
Heart problems (%)				.67^e^			.05^e^				.37^e^
No	4,832 (88.9)	1,731 (88.6)	3,101 (89.0)		1,058 (90.5)	3,774 (88.4)		804 (90.1)	1,181 (88.3)	2,847 (88.8)	
Yes	605 (11.1)	222 (11.4)	383 (11.0)		111 (9.5)	494 (11.6)		88 (9.9)	157 (11.7)	360 (11.2)	
Stroke (%)				.03^e^			.34^e^				.05^e^
No	5,335 (98.1)	1,927 (98.7)	3,408 (97.8)		1,151 (98.5)	4,184 (98.0)		878 (98.4)	1,322 (98.8)	3,135 (97.8)	
Yes	102 (1.9)	26 (1.3)	76 (2.2)		18 (1.5)	84 (2.0)		14 (1.6)	16 (1.2)	72 (2.2)	
Arthritis or rheumatism (%)				<.01^e^			.03^e^				<.01^e^
No	3,568 (65.6)	1,337 (68.5)	2,231 (64.0)		799 (68.3)	2,769 (64.9)		625 (70.1)	886 (66.2)	2,057 (64.1)	
Yes	1,869 (34.4)	616 (31.5)	1,253 (36.0)		370 (31.7)	1,499 (35.1)		267 (29.9)	452 (33.8)	1,150 (35.9)	
Liver diseases (%)				.58^e^			.96^e^				.91^e^
No	5,266 (96.9)	1,895 (97.0)	3,371 (96.8)		1,132 (96.8)	4,134 (96.9)		866 (97.1)	1,295 (96.8)	3,105 (96.8)	
Yes	171 (3.1)	58 (3.0)	113 (3.2)		37 (3.2)	134 (3.1)		26 (2.9)	43 (3.2)	102 (3.2)	
Kidney diseases (%)				.03^e^			.35^e^				.04^e^
No	5,142 (94.6)	1,864 (95.4)	3,278 (94.1)		1,112 (95.1)	4,030 (94.4)		847 (95.0)	1,282 (95.8)	3,013 (94.0)	
Yes	295 (5.4)	89 (4.6)	206 (5.9)		57 (4.9)	238 (5.6)		45 (5.0)	56 (4.2)	194 (6.0)	
Digestive diseases (%)				<.01^e^			.19^e^				<.01^e^
No	4,215 (77.5)	1,570 (80.4)	2,645 (75.9)		923 (79.0)	3,292 (77.1)		711 (79.7)	1,071 (80.0)	2,433 (75.9)	
Yes	1,222 (22.5)	383 (19.6)	839 (24.1)		246 (21.0)	976 (22.9)		181 (20.3)	267 (20.0)	774 (24.1)	
Asthma (%)				<.01^e^			.09^e^				<.01^e^
No	5,187 (95.4)	1,885 (96.5)	3,302 (94.8)		1,126 (96.3)	4,061 (95.1)		869 (97.4)	1,273 (95.1)	3,045 (94.9)	
Yes	250 (4.6)	68 (3.5)	182 (5.2)		43 (3.7)	207 (4.9)		23 (2.6)	65 (4.9)	162 (5.1)	
Memory-related diseases (%)				.10^e^			.05^e^				.09^e^
No	5,375 (98.9)	1,937 (99.2)	3,438 (98.7)		1,162 (99.4)	4,213 (98.7)		886 (99.3)	1,327 (99.2)	3,162 (98.6)	
Yes	62 (1.1)	16 (.8)	46 (1.3)		7 (.6)	55 (1.3)		6 (.7)	11 (.8)	45 (1.4)	
Number of chronic condition (%)				<.01^e^			.05^e^				<.02^e^
0	1,846 (34.0)	695 (35.6)	1,151 (33.0)		402 (34.4)	1,444 (33.8)		316 (35.4)	465 (34.8)	1,065 (33.2)	
1	1,719 (31.6)	637 (32.6)	1,082 (31.1)		397 (34.0)	1,322 (31.0)		311 (34.9)	412 (30.8)	996 (31.1)	
≥2	1,872 (34.4)	621 (31.8)	1,251 (35.9)		370 (31.7)	1,502 (35.2)		265 (29.7)	461 (34.5)	1,146 (35.7)	

^a^ Compare clean fuel and solid fuel for cooking.

^b^ Compare clean fuel and solid fuel for heating.

^c^ Compare clean fuel for both, solid fuel for either, and solid fuel for both cooking and heating.

^d^ Analysis of Student’s t-test or Analysis of Variance (ANOVA).

^e^ Analysis of Chi-square test.

**TABLE 2:** Baseline characteristics of participants according to the progression of multimorbidity during follow-up (China Health and Retirement Longitudinal Study, China, 2011–2018).

Characteristics	Total	Stable condition progression	Single condition progression	Multimorbidity progression	*p*-value^a^
No (%)	5,437 (100)	1,806 (33.2)	620 (11.4)	3,011 (55.4)	
Age (mean ± [SD])	57.9 ± 8.5	56.9 ± 8.4	56.7 ± 8.9	58.8 ± 8.5	<.01^b^
Sex (%)					.20^c^
Male	2,541 (46.7)	859 (47.6)	305 (49.2)	1,377 (45.7)	
Female	2,896 (53.3)	947 (52.4)	315 (50.8)	1,634 (54.3)	
Education (%)					.20^c^
Less than lower secondary	4,959 (91.2)	1,626 (90.0)	572 (92.3)	2,761 (91.7)	
Upper secondary & vocational training	446 (8.2)	168 (9.3)	43 (6.9)	235 (7.8)	
Tertiary	32 (.6)	12 (.7)	5 (.8)	15 (.5)	
Marital status (%)					.17^c^
Married/partnered	4,930 (90.7)	1,653 (91.5)	567 (91.5)	2,710 (90.0)	
Others	507 (9.3)	153 (8.5)	53 (8.5)	301 (10.0)	
Working status (%)					<.01^c^
Unemployed/retired/never worked	1,586 (29.2)	482 (26.7)	148 (23.9)	956 (31.8)	
Employed	3,851 (70.8)	1,324 (73.3)	472 (76.1)	2,055 (68.2)	
Residence (%)					.99^c^
Urban	1,624 (29.9)	539 (29.8)	184 (29.7)	901 (29.9)	
Rural	3,813 (70.1)	1,267 (70.2)	436 (70.3)	2,110 (70.1)	
Household income (%)					.41^c^
Quartile 1 (lowest)	1,360 (25.0)	434 (24.0)	178 (28.7)	748 (24.8)	
Quartile 2	1,359 (25.0)	460 (25.5)	141 (22.7)	758 (25.2)	
Quartile 3	1,360 (25.0)	458 (25.4)	154 (24.8)	748 (24.8)	
Quartile 4 (highest)	1,358 (25.0)	454 (25.1)	147 (23.7)	757 (25.1)	
Smoking status (%)					.43^c^
Ever/never smoking	3,727 (68.5)	1,221 (67.6)	420 (67.7)	2,086 (69.3)	
Current smoking	1,710 (31.5)	585 (32.4)	200 (32.3)	925 (30.7)	
Drinking status (%)					<.01^c^
Never drinking	3,624 (66.7)	1,165 (64.5)	390 (62.9)	2,069 (68.7)	
Ever drinking	1,813 (33.3)	641 (35.5)	230 (37.1)	942 (31.3)	
BMI (%)					<.01^c^
Underweight	358 (6.6)	119 (6.6)	34 (5.5)	205 (6.8)	
Normal weight	2,900 (53.3)	1,045 (57.9)	377 (60.8)	1,478 (49.1)	
Overweight	1,552 (28.5)	474 (26.2)	156 (25.2)	922 (30.6)	
Obese	627 (11.5)	168 (9.3)	53 (8.5)	406 (13.5)	
Cooking fuel (%)					<.01^c^
Clean fuel	1,953 (35.9)	705 (39.0)	227 (36.6)	1,021 (33.9)	
Solid fuel	3,484 (64.1)	1,101 (61.0)	393 (63.4)	1,990 (66.1)	
Heating fuel (%)					<.01^c^
Clean fuel	1,169 (21.5)	448 (24.8)	124 (20.0)	597 (19.8)	
Solid fuel	4,268 (78.5)	1,358 (75.2)	496 (80.0)	2,414 (80.2)	
Cooking and heating fuel (%)					<.01^c^
Both clean fuel	892 (16.4)	340 (18.8)	93 (15.0)	459 (15.2)	
Either for solid fuel	1,338 (24.6)	473 (26.2)	165 (26.6)	700 (23.2)	
Both solid fuel	3,207 (59.0)	993 (55.0)	362 (58.4)	1,852 (61.5)	

^a^ Compare stable condition progression, single condition progression, and multimorbidity progression.

^b^ Analysis of ANOVA.

^c^ Analysis of Chi-square test.

### Solid Fuel Use and Multimorbidity

Solid fuel use was associated with the progression of multimorbidity (Table 3). Solid fuel use for cooking or heating was associated with multimorbidity progression with adjusted odds of 1.16 (95% CI: 1.01–1.34) or 1.28 (95% CI: 1.09–1.50), respectively, compared with clean fuel use. Both solid fuel use for cooking and heating was also associated with multimorbidity progression with adjusted odds of 1.42 (95% CI: 1.19–1.70), compared with both clean fuel use for cooking and heating.

**TABLE 3:** Associations of solid fuel use with the progression of multimorbidity during follow-up (China Health and Retirement Longitudinal Study, China, 2011–2018).

	Stable condition progression	Single condition progression			Multimorbidity progression		
		Model 1	Model 2	Model 3	Model 1	Model 2	Model 3
Cooking^a^							
Clean fuel	Ref	Ref	Ref	Ref	Ref	Ref	Ref
Solid fuel	Ref	1.00 (.81–1.23)^b^	.98 (.79–1.22)	.98 (.79–1.22)	1.15 (1.00–1.32)	1.15 (1.00–1.32)	1.16 (1.01–1.34)
Heating^c^							
Clean fuel	Ref	Ref	Ref	Ref	Ref	Ref	Ref
Solid fuel	Ref	1.32 (1.03–1.69)	1.33 (1.03–1.72)	1.34 (1.03–1.73)	1.24 (1.06–1.45)	1.28 (1.09–1.50)	1.28 (1.09–1.50)
Cooking and heating^d^							
Both clean fuel	Ref	Ref	Ref	Ref	Ref	Ref	Ref
Either solid fuel	Ref	1.28 (.95–1.70)	1.30 (.96–1.74)	1.30 (.96–1.75)	1.10 (.91–1.32)	1.11 (.92–1.34)	1.10 (.91–1.32)
Both solid fuel	Ref	1.33 (1.03–1.73)	1.33 (1.00–1.76)	1.33 (1.00–1.77)	1.38 (1.18–1.62)	1.42 (1.19–1.69)	1.42 (1.19–1.70)

^a^ Compare clean fuel and solid fuel for cooking.

^b^ Odds ratio (95% confidence interval) (all such value).

^c^ Compare clean fuel and solid fuel for heating.

^d^ Compare clean fuel for both, solid fuel for either, and solid fuel for both cooking and heating.

Model 1 was unadjusted.

Model 2 was adjusted for age, sex, education levels, marital status, working status, household income, and residence.

Model 3 was adjusted for age, sex, education levels, marital status, working status, household income, residence, smoking status, drinking status, and BMI.

### Additional Analyses

Use of solid fuel for both cooking and heating was associated with most of the 12 single chronic conditions such as chronic lung diseases (adjusted OR: 1.38, 95% CI: 1.06–1.79), arthritis (adjusted OR: 1.42, 95% CI: 1.14–1.78), and memory-related diseases (adjusted OR: 2.30, 95% CI: 1.44–3.68), compared with clean fuel use for both (Supplementary Table S3). In the subgroup analyses (Supplementary Table S5), solid fuel use for both cooking and heating was associated with multimorbidity progression in urban (adjusted OR: 1.47, 95% CI: 1.12–1.93) and rural (adjusted OR: 1.38, 95% CI: 1.08–1.75), participants without multimorbidity at baseline (adjusted OR: 1.42, 95% CI: 1.05–1.91), and participants with multimorbidity at baseline (adjusted OR: 1.53, 95% CI: 1.13–2.08), compared with clean fuel use for both. The results from sensitivity analyses were similar to the main analysis (Supplementary Tables S6–S8). For example, solid fuel use for both cooking and heating was also associated with the progression of multimorbidity in participants without the selected conditions at baseline (adjusted OR: 1.28, 95% CI: .90–1.81) and defining electric or solar as clean fuel (adjusted OR: 1.41, 95% CI: 1.09–1.82), compared with clean fuel use for both. The participant who used 7 years or more of solid fuel for cooking was associated with multimorbidity progression (adjusted OR: 1.36, 95% CI: 1.13–1.62), compared with 0 years of solid fuel use for cooking.

## Discussion

Based on the nationally representative cohort study of CHARLS, this study found that using solid fuels was significantly associated with the progression of multimorbidity, compared with clean fuel use. This highlights the role of household air pollution in controlling and preventing multimorbidity.

First, this study found that solid fuel use for cooking, heating, and for both cooking and heating were associated with multimorbidity progression, compared with clean fuel use, respectively. Our findings were consistent with a study that reported that solid fuel use was associated with the incidence of cardiometabolic multimorbidity (CMM) (hazard ratio [HR]: 1.71,95% CI: 1.28, 2.28) [29]. Our study added evidence on the disease burden of household air pollution, where previous studies had only estimated the disease-specific relative risk (RR) of single conditions like asthma (RR:1.23, 95% CI: 1.11–1.36) [12], and our study also highlighted the importance of reducing solid fuel use to prevent multimorbidity. However, only one previous study reported the association between solid fuel use and multimorbidity. Considering that people were more likely to develop multimorbidity as the population aged, 49.64% of the elderly in China were estimated to have multimorbidity in 2015 [16, 30]. Most previous studies also reported that people with multimorbidity were at higher risk of death, had a longer hospital stay, had a poorer quality of life, and had poorer physical function than those with a single chronic condition [31–33]. Therefore, more longitudinal and representative cohort studies were needed to explore risk factors of multimorbidity, providing more evidence about multimorbidity prevention for policymakers.

Second, this study identified that the association of solid fuel use for heating with the progression of multimorbidity was stronger than solid fuel use for cooking. One explanation for this result was that the utilization rate of solid fuels for heating was higher than that for cooking in this study (78.5% vs. 64.1%). Another potential reason was the different exposure patterns from cooking and heating that burning solid fuel for heating might be kept going all day in the winter months, often with poor ventilation, whereas cooking is kept going several times per day. Moreover, according to previous research [34, 35], burning solid fuel for heating creates more pollutants and a longer length of exposure than cooking, which may explain the higher association with the progression of multimorbidity. The burden of household air pollution remained high in the world’s regions contributing to 2.31 million deaths and 91.5 million DALYs in 2019 [36]. Considering solid fuel use for cooking and heating were two exposure patterns, leading to different health impacts on people, separate interventions for solid fuel use for cooking and heating were needed to reduce the adverse health impacts and burden of household air pollution effectively.

Third, this study also found that the joint effect of solid fuel use for both cooking and heating was stronger in urban than rural areas. The first explanation for this result was that in this study, the prevalence of multimorbidity at baseline was higher in urban than rural areas (34.5% vs. 34.4%) and participants with multimorbidity at baseline demonstrated a higher association between solid fuel use and multimorbidity progression than those without multimorbidity at baseline. It was consistent with previous studies which reported that people had a higher cumulative incidence of multimorbidity among those with the presence of ≥2 chronic conditions at baseline [37, 38]. The second explanation was that the prevalence of obesity in urban areas was higher than in rural areas (15.6% vs. 9.8%) in this study, many previous studies also reported that obesity was the risk factor for multimorbidity [39–41]. Another potential reason was that people living in urban areas were more likely to be exposed to outdoor air pollution such as urban traffic. Luo and his colleagues have reported that outdoor air pollution was associated with the development of CMM [42]. However, the information on outdoor air pollution was not collected in this study. Future research should consider this factor, which could explain the different relationships of solid fuel use and multimorbidity progression between urban and rural areas specifically.

### Limitations and Strengths

The present study had some strengths. First, we provided prospective evidence on the association between solid fuel use and multimorbidity based on a relatively large sample size in a nationally representative cohort study in China. Second, the separate roles of solid fuel use for cooking and heating on multimorbidity were examined. Third, the sensitivity and subgroup analyses have confirmed the robustness of our results. Our study has some limitations that should be noted. First, as in other studies [29], information on solid fuel use at baseline was used to explore the association with multimorbidity progression without considering the changes in solid fuel use during follow-up. But we did the sensitivity analysis of estimating the association between the duration of solid fuel use for cooking from 2011 to 2018 and multimorbidity progression, which was consistent with our result. Second, as in other studies [43–45], the self-report use of solid fuel may be affected by reporting bias and using self-reported fuel use as a proxy for household air pollution without the levels of exposure separated by frequency of use of the solid fuel may not fully capture the extent of one’s exposure. Third, missing data on solid fuel use, chronic conditions, and covariates were excluded from this study and may be affected by select bias. Fourth, the information on seasonal, altitude, and temperature variations in the areas of the study, ventilation status when using solid fuel, and solid fuel use in the past which may relate to the exposure to household air pollution were not collected in this cohort study. We could not identify the influence of these factors on the association between solid fuel use and multimorbidity progression. Fifth, despite adjusting several covariates in our analyses, residual confounding remains a possibility.

### Conclusion

This study showed that solid fuel use was associated with the progression of multimorbidity. Our findings indicate that reducing solid fuel use and promoting clean fuel use should be integrated into household air pollution for the control and the prevention of multimorbidity.
