# Supplementary material for: Solid Fuel Use and the Progression of Multimorbidity in Middle-Aged Chinese Participants: A Prospective Cohort Study
Source: Int J Public Health. 2023 Jan 9;67:1605206. doi: 10.3389/ijph.2022.1605206 (PMC9880982; doi:10.3389/ijph.2022.1605206)
Supplement: Supplementary file 1 [file DataSheet1.docx]

International Journal of Public Health

Solid fuel use and the progression of multimorbidity in middle-aged Chinese participants: a prospective cohort study

Table S1. Baseline characteristics according to the sample excluding or not excluding missing data (China Health and Retirement Longitudinal Study, China, 2011).

| **Characteristics** | **Excluded** | **Non-excluded** | **P value^*^** |
| --- | --- | --- | --- |
|  | (N = 5,437) | (N = 11,442) |  |
| **Age (mean ± standard deviation[SD])** | 57.9 ± 8.5 | 58.2 ± 8.8 | 0.25^a^ |
| **Sex (%)** |  |  | 0.52^b^ |
| Male | 2,541 (46.7) | 5,408 (47.3) |  |
| Female | 2,896 (53.3) | 6,034 (52.7) |  |
| **Education (%)** |  |  | < 0.01^b^ |
| Less than lower secondary | 4,959 (91.2) | 10,228 (89.4) |  |
| Upper secondary & vocational training | 446 (8.2) | 1,082 (9.5) |  |
| Tertiary | 32 (0.6) | 132 (1.2) |  |
| **Marital status (%)** |  |  | < 0.01^b^ |
| Married/partnered | 4,930 (90.7) | 10,216 (89.3) |  |
| Others | 507 (9.3) | 1,223 (10.7) |  |
| **Working status (%)** |  |  | < 0.01^b^ |
| Unemployed/retired/never worked | 1,586 (29.2) | 3,567 (31.8) |  |
| Employed | 3,851 (70.8) | 7,665 (68.2) |  |
| **Residence (%)** |  |  | < 0.01^b^ |
| Urban | 1,624 (29.9) | 3,961 (34.6) |  |
| Rural | 3,813 (70.1) | 7,481 (65.4) |  |
| **Household income (%)** |  |  | 1.00^b^ |
| Quartile 1 (lowest) | 1,360 (25.0) | 2,424 (25.0) |  |
| Quartile 2 | 1,359 (25.0) | 2,421 (25.0) |  |
| Quartile 3 | 1,360 (25.0) | 2,423 (25.0) |  |
| Quartile 4 (highest) | 1,358 (25.0) | 2,421 (25.0) |  |
| **Smoking status (%)** |  |  | 0.02^b^ |
| Ever/never smoking | 3,727 (68.5) | 7,786 (70.3) |  |
| Current smoking | 1,710 (31.5) | 3,292 (29.7) |  |
| **Drinking status (%)** |  |  | 0.90^b^ |
| Never drinking | 3,624 (66.7) | 7,580 (66.6) |  |
| Ever drinking | 1,813 (33.3) | 3,809 (33.4) |  |
| **BMI (%)** |  |  | 0.86^b^ |
| Underweight | 358 (6.6) | 605 (6.4) |  |
| Normal weight | 2,900 (53.3) | 4,985 (52.8) |  |
| Overweight | 1,552 (28.5) | 2,740 (29.0) |  |
| Obese | 627 (11.5) | 1,107 (11.7) |  |
| **Cooking fuel (%)** |  |  | < 0.01^b^ |
| Clean fuel | 1,953 (35.9) | 4,769 (42.3) |  |
| Solid fuel | 3,484 (64.1) | 6,513 (57.7) |  |
| **Heating fuel (%)** |  |  | 0.02^b^ |
| Clean fuel | 1,169 (21.5) | 1,982 (23.2) |  |
| Solid fuel | 4,268 (78.5) | 6,552 (76.8) |  |
| **Hypertension (%)** |  |  | 0.27^b^ |
| No | 4,124 (75.9) | 8,508 (75.1) |  |
| Yes | 1,313 (24.1) | 2,825 (24.9) |  |
| **Diabetes (%)** |  |  | 0.17^b^ |
| No | 5,151 (94.7) | 10,637 (94.2) |  |
| Yes | 286 (5.3) | 652 (5.8) |  |
| **Cancer (%)** |  |  | 0.35^b^ |
| No | 5,402 (99.4) | 11,248 (99.2) |  |
| Yes | 35 (0.6) | 88 (0.8) |  |
| **Lung diseases (%)** |  |  | 0.33^b^ |
| No | 4,926 (90.6) | 10,333 (91.1) |  |
| Yes | 511 (9.4) | 1,014 (8.9) |  |
| **Heart problems (%)** |  |  | 0.45^b^ |
| No | 4,832 (88.9) | 10,021 (88.5) |  |
| Yes | 605 (11.1) | 1,305 (11.5) |  |
| **Stroke (%)** |  |  | 0.21^b^ |
| No | 5,335 (98.1) | 11,116 (97.8) |  |
| Yes | 102 (1.9) | 247 (2.2) |  |
| **Arthritis or rheumatism (%)** |  |  | 0.71^b^ |
| No | 3,568 (65.6) | 7,492 (65.9) |  |
| Yes | 1,869 (34.4) | 3,874 (34.1) |  |
| **Liver diseases (%)** |  |  | 0.33^b^ |
| No | 5,266 (96.9) | 10,919 (96.6) |  |
| Yes | 171 (3.1) | 388 (3.4) |  |
| **Kidney diseases (%)** |  |  | 0.81^b^ |
| No | 5,142 (94.6) | 10,691 (94.5) |  |
| Yes | 295 (5.4) | 624 (5.5) |  |
| **Digestive diseases (%)** |  |  | 0.98^b^ |
| No | 4,215 (77.5) | 8,805 (77.5) |  |
| Yes | 1,222 (22.5) | 2,555 (22.5) |  |
| **Asthma (%)** |  |  | 0.23^b^ |
| No | 5,187 (95.4) | 10,869 (95.8) |  |
| Yes | 250 (4.6) | 476 (4.2) |  |
| **Memory-related diseases (%)** |  |  | 0.18^b^ |
| No | 5,375 (98.9) | 11,194 (98.6) |  |
| Yes | 62 (1.1) | 158 (1.4) |  |

^*^Compare the sample excluding or not excluding missing data.

^a^ Analysis of Students’t test.

^b^ Analysis of Chi-square test.

Table S2. Characteristics of all variables were presented from wave 2011 to wave 2018 (N = 5,437) (China Health and Retirement Longitudinal Study, China, 2011 - 2018).

| **Characteristics** | **Wave 2011** | **Wave 2013** | **Wave 2015** | **Wave 2018** |
| --- | --- | --- | --- | --- |
| **Age (mean ± [SD])** | 57.9 ± 8.5 | 60.0 ± 8.6 | 61.9 ± 8.6 | 64.9 ± 8.6 |
| **Sex (%)** |  |  |  |  |
| Male | 2,541 (46.7) | 2,541 (46.7) | 2,541 (46.7) | 2,541 (46.7) |
| Female | 2,896 (53.3) | 2,896 (53.3) | 2,896 (53.3) | 2,896 (53.3) |
| **Education (%)** |  |  |  |  |
| Less than lower secondary | 4,959 (91.2) | 4,959 (91.2) | 4,959 (91.2) | 4,959 (91.2) |
| Upper secondary & vocational training | 446 (8.2) | 446 (8.2) | 446 (8.2) | 446 (8.2) |
| Tertiary | 32 (0.6) | 32 (0.6) | 32 (0.6) | 32 (0.6) |
| **Marital status (%)** |  |  |  |  |
| Married/partnered | 4,930 (90.7) | 4,875 (89.7) | 4,764 (87.6) | 4,553 (83.7) |
| Others | 507 (9.3) | 562 (10.3) | 673 (12.4) | 884 (16.3) |
| **Working status (%)** |  |  |  |  |
| Unemployed/retired/never worked | 1,586 (29.2) | 1,518 (28.0) | 1,753 (32.3) | 2,022 (37.2) |
| Employed | 3,851 (70.8) | 3,913 (72.0) | 3,674 (67.7) | 3,413 (62.8) |
| **Residence (%)** |  |  |  |  |
| Urban | 1,624 (29.9) | 1,624 (29.9) | 1,624 (29.9) | 1,624 (29.9) |
| Rural | 3,813 (70.1) | 3,813 (70.1) | 3,813 (70.1) | 3,813 (70.1) |
| **Household income (Median [inter-quartile range)** | 15,346 (16,360) | 21,830 (23,708) | 22,540 (26,210) | 23,960 (29,388) |
| **Smoking status (%)** |  |  |  |  |
| Ever/never smoking | 3,727 (68.6) | 3,466 (82.5) | 3,885 (71.5) | 3,985 (73.3) |
| Current smoking | 1,710 (31.5) | 7,36 (17.5) | 1,549 (28.5) | 1,451 (26.7) |
| **Drinking status (%)** |  |  |  |  |
| Never drinking | 3,624 (66.7) | 3,599 (66.2) | 3,632 (66.8) | 3,776 (69.5) |
| Ever drinking | 1,813 (33.4) | 1,834 (33.2) | 1,804 (33.2) | 1,660 (30.5) |
| **BMI (mean ± [SD])** | 2.5 ± 0.8 | 2.5 ± 0.8 | 2.5 ± 0.8 | / |
| **Cooking fuel (%)** |  |  |  |  |
| Clean fuel | 1,953 (35.9) | 2,450 (45.3) | 2,637 (49.1) | 3,189 (59.9) |
| Solid fuel | 3,484 (64.1) | 2,961 (54.7) | 2,738 (50.9) | 2,131 (40.1) |
| **Heating fuel (%)** |  |  |  |  |
| Clean fuel | 1,169 (21.5) | 972 (22.4) | / | / |
| Solid fuel | 4,268 (78.5) | 3,367 (77.6) | / | / |
| **Hypertension (%)** |  |  |  |  |
| No | 3,205 (76.1) | 3,943 (72.5) | 3,592 (66.1) | 2,998 (55.1) |
| Yes | 1,009 (23.9) | 1,494 (27.5) | 1,845 (33.9) | 2,439 (44.9) |
| **Diabetes (%)** |  |  |  |  |
| No | 4,007 (95.1) | 5,077 (93.4) | 4,932 (90.7) | 4,628 (85.1) |
| Yes | 207 (4.9) | 360 (6.6) | 505 (9.3) | 809 (14.9) |
| **Cancer (%)** |  |  |  |  |
| No | 4,189 (99.4) | 5,394 (99.2) | 5,365 (98.7) | 5,292 (97.3) |
| Yes | 25 (0.6) | 43 (0.8) | 72 (1.3) | 145 (2.7) |
| **Lung diseases (%)** |  |  |  |  |
| No | 3,829 (90.9) | 4,861 (89.4) | 4,657 (85.7) | 4,251 (78.2) |
| Yes | 385 (9.1) | 576 (10.6) | 780 (14.3) | 1,186 (21.8) |
| **Heart problems (%)** |  |  |  |  |
| No | 3,753 (89.1) | 4,727 (86.9) | 4,473 (82.3) | 4,095 (75.3) |
| Yes | 461 (10.9) | 710 (13.1) | 964 (17.7) | 1,342 (24.7) |
| **Stroke (%)** |  |  |  |  |
| No | 4,134 (98.1) | 5,311 (97.7) | 5,253 (96.6) | 4,945 (91.0) |
| Yes | 80 (1.9) | 126 (2.3) | 184 (3.4) | 492 (9.0) |
| **Arthritis or rheumatism (%)** |  |  |  |  |
| No | 2,761 (65.5) | 3,441 (63.3) | 2,905 (53.4) | 2,546 (46.8) |
| Yes | 1,453 (34.5) | 1,996 (36.7) | 2,532 (46.6) | 2,891 (53.2) |
| **Liver diseases (%)** |  |  |  |  |
| No | 4,089 (97.0) | 5,220 (96.0) | 5,098 (93.8) | 4,918 (90.5) |
| Yes | 125 (3.0) | 217 (4.0) | 339 (6.2) | 519 (9.5) |
| **Kidney diseases (%)** |  |  |  |  |
| No | 3,980 (94.4) | 5,069 (93.2) | 4,908 (90.3) | 4,698 (86.4) |
| Yes | 234 (5.6) | 368 (6.8) | 529 (9.7) | 739 (13.6) |
| **Digestive diseases (%)** |  |  |  |  |
| No | 3,250 (77.1) | 4,095 (75.3) | 3,645 (67.0) | 3,320 (61.1) |
| Yes | 964 (22.9) | 1,342 (24.7) | 1,792 (33.0) | 2,117 (38.9) |
| **Asthma (%)** |  |  |  |  |
| No | 4,025 (95.5) | 5,172 (95.1) | 5,088 (93.6) | 4,970 (91.4) |
| Yes | 189 (4.5) | 265 (4.9) | 349 (6.4) | 467 (8.6) |
| **Memory-related diseases (%)** |  |  |  |  |
| No | 4,169 (98.9) | 5,360 (98.6) | 5,315 (97.8) | 5,164 (95.0) |
| Yes | 45 (1.1) | 77 (1.4) | 122 (2.2) | 273 (5.0) |

Table S3. Associations of the joint of solid fuel use for cooking and heating with the incidence of 12 single chronic conditions with the fully adjusted model (N = 5,437) (China Health and Retirement Longitudinal Study, China, 2011 - 2018).

|  | **Both clean fuel use** | **Either solid fuel use** | **Both solid fuel use** |
| --- | --- | --- | --- |
| **Hypertension** |  |  |  |
| No | Ref | Ref | Ref |
| Yes | Ref | 0.85 (0.68 - 1.08) ^a^ | 1.14 (0.92 - 1.41) |
| **Diabetes** |  |  |  |
| No | Ref | Ref | Ref |
| Yes | Ref | 0.79 (0.59 - 1.07) | 0.96 (0.73 - 1.27) |
| **Cancer** |  |  |  |
| No | Ref | Ref | Ref |
| Yes | Ref | 1.29 (0.69 - 2.40) | 1.55 (0.87 - 2.76) |
| **Lung diseases** |  |  |  |
| No | Ref | Ref | Ref |
| Yes | Ref | 1.12 (0.84 - 1.49) | 1.38 (1.06 - 1.79) |
| **Heart problems** |  |  |  |
| No | Ref | Ref | Ref |
| Yes | Ref | 1.76 (1.34 - 2.32) | 1.68 (1.29 - 2.19) |
| **Stroke** |  |  |  |
| No | Ref | Ref | Ref |
| Yes | Ref | 1.30 (0.91 - 1.87) | 1.46 (1.04 - 2.05) |
| **Arthritis or rheumatism** |  |  |  |
| No | Ref | Ref | Ref |
| Yes | Ref | 1.06 (0.83 - 1.35) | 1.42 (1.14 - 1.78) |
| **Liver diseases** |  |  |  |
| No | Ref | Ref | Ref |
| Yes | Ref | 0.61 (0.43 - 0.87) | 0.94 (0.70 - 1.28) |
| **Kidney diseases** |  |  |  |
| No | Ref | Ref | Ref |
| Yes | Ref | 1.03 (0.74 - 1.44) | 1.30 (0.96 - 1.76) |
| **Digestive diseases** |  |  |  |
| No | Ref | Ref | Ref |
| Yes | Ref | 0.92 (0.73 - 1.17) | 1.02 (0.82 - 1.28) |
| **Asthma** |  |  |  |
| No | Ref | Ref | Ref |
| Yes | Ref | 0.99 (0.61 - 1.59) | 1.20 (0.78 - 1.85) |
| **Memory-related diseases** |  |  |  |
| No | Ref | Ref | Ref |
| Yes | Ref | 1.66 (1.00 - 2.75) | 2.30 (1.44 - 3.68) |

Model 3 was adjusted for age, sex, education levels, marital status, working status, household income, residence, smoking status, drinking status, and BMI.

^a^ Odds ratio (95% confidence interval) (all such value).

Table S4. Associations between specific types of solid fuel use for cooking and heating and the progression of multimorbidity with the fully adjusted model (N = 5,437) (China Health and Retirement Longitudinal Study, China, 2011 - 2018).

|  | **Case of multimorbidity progression/number.(%)** | **Stable condition progression** | **Single condition progression** | **Multimorbidity progression** |
| --- | --- | --- | --- | --- |
| **Cooking fuel** |  |  |  |  |
| Electric | 470/889 (52.9) | Ref | Ref | Ref |
| Coal | 443/779 (56.9) | Ref | 1.02 (0.72 - 1.44)^a^ | 1.13 (0.90 - 1.41) |
| Natural gas | 181/337 (53.7) | Ref | 0.96 (0.60 - 1.53) | 1.01 (0.75 - 1.36) |
| Marsh gas | 39/75 (52.0) | Ref | 0.93 (0.42 - 2.05) | 1.10 (0.65 - 1.85) |
| Liquefied petroleum gas | 331/652 (50.8) | Ref | 0.99 (0.70 - 1.40) | 0.91 (0.72 - 1.14) |
| Crop residue/wood | 1,547/2,705 (57.2) | Ref | 0.95 (0.72 - 1.27) | 1.10 (0.91 - 1.32) |
| **Heating fuel** |  |  |  |  |
| Electric | 417/820 (50.9) | Ref | Ref | Ref |
| Solar | 74/142 (52.1) | Ref | 0.59 (0.29 - 1.21) | 1.13 (0.90 - 1.41) |
| Coal | 1,223/2,230 (54.8) | Ref | 1.27 (0.94 - 1.70) | 1.18 (0.98 - 1.43) |
| Natural gas | 51/104 (49.0) | Ref | 1.19 (0.57 - 2.46) | 0.81 (0.50 - 1.32) |
| Liquefied petroleum gas | 55/104 (52.9) | Ref | 0.99 (0.48 - 2.06) | 1.24 (0.79 - 1.96) |
| Crop residue/wood | 1,191/2,038 (58.4) | Ref | 1.30 (0.94 - 1.80) | 1.42 (1.16 - 1.75) |

Model 3 was adjusted for age, sex, education levels, marital status, working status, household income, residence, smoking status, drinking status, and BMI.

^a^ Odds ratio (95% confidence interval) (all such value).

Table S5 Subgroup analysis for associations of the joint of solid fuel use for cooking and heating with the progression of multimorbidity with the fully adjusted model (n = 3,011) (China Health and Retirement Longitudinal Study, China, 2011 - 2018).

|  | **Multimorbidity progression** | | |
| --- | --- | --- | --- |
| **Characteristics** | **Both clean fuel use** | **Either solid fuel use** | **Both solid fuel use** |
| **Age** |  |  |  |
| < 65 | Ref | 1.10 (0.89 - 1.35)^a^ | 1.49 (1.23 - 1.80) |
| ≥ 65 | Ref | 1.18 (0.73 - 1.90) | 1.17 (0.76 - 1.82) |
| **Sex** |  |  |  |
| Male | Ref | 1.01 (0.77 - 1.33) | 1.39 (1.07 - 1.79) |
| Female | Ref | 1.21 (0.93 - 1.57) | 1.46 (1.15 - 1.86) |
| **Education** |  |  |  |
| Less than tertiary | Ref | 1.08 (0.89 - 1.33) | 1.40 (1.16 - 1.69) |
| Tertiary | Ref | 1.32 (0.77 - 2.25) | 1.62 (0.92 - 2.82) |
| **Marital status** |  |  |  |
| Married/partnered | Ref | 1.06 (0.87 - 1.29) | 1.39 (1.15 - 1.67) |
| Others | Ref | 1.85 (0.96 - 3.57) | 1.92 (1.07 - 3.47) |
| **Residence** |  |  |  |
| Urban | Ref | 1.09 (0.84 - 1.43) | 1.47 (1.12 - 1.93) |
| Rural | Ref | 1.09 (0.83 - 1.43) | 1.38 (1.08 - 1.75) |
| **Working status** |  |  |  |
| Unemployed/retired/never worked | Ref | 1.22 (0.88 - 1.69) | 1.62 (1.18 - 2.21) |
| Employed | Ref | 1.05 (0.83 - 1.32) | 1.34 (1.08 - 1.66) |
| **Household income** |  |  |  |
| Quartile 1 (lowest) and 2 | Ref | 1.16 (0.83 - 1.60) | 1.36 (1.00 - 1.85) |
| Quartile 3 and 4 (highest) | Ref | 1.06 (0.84 - 1.33) | 1.47 (1.18 - 1.83) |
| **Smoking status** |  |  |  |
| Ever/never smoking | Ref | 1.15 (0.92 - 1.44) | 1.48 (1.20 - 1.83) |
| Current smoking | Ref | 1.01 (0.72 - 1.43) | 1.30 (0.94 - 1.80) |
| **Drinking status** |  |  |  |
| Never drinking | Ref | 1.18 (0.93 - 1.49) | 1.45 (1.17 - 1.80) |
| Ever drinking | Ref | 0.97 (0.71 - 1.33) | 1.35 (1.00 - 1.82) |
| **BMI** |  |  |  |
| Non-obese | Ref | 1.06 (0.87 - 1.29) | 1.42 (1.18 - 1.71) |
| Obese | Ref | 1.65 (0.94 - 2.90) | 1.46 (0.86 - 2.47) |
| **Multimorbidity status at baseline** |  |  |  |
| No | Ref | 1.05 (0.83 - 1.34) | 1.42 (1.05 - 1.91) |
| Yes | Ref | 1.10 (0.80 - 1.52) | 1.53 (1.13 - 2.08) |

Model 3 was adjusted for age, sex, education levels, marital status, working status, household income, residence, smoking status, drinking status, and BMI.

^a^ Odds ratio (95% confidence interval) (all such value).

Table S6. Sensitivity analysis for associations of the joint of solid fuel use for cooking and heating with the progression of multimorbidity during follow-up in participants without selected chronic conditions at baseline with the fully adjusted model (n = 1,837) (China Health and Retirement Longitudinal Study, China, 2011 - 2018).

|  | **Case of multimorbidity progression/Number (%)** | **Stable condition progression** | **Single condition progression** | **Multimorbidity progression** |
| --- | --- | --- | --- | --- |
| **Cooking and heating** |  |  |  |  |
| Both clean fuel use | 94/316 (29.7) | Ref | Ref | Ref |
| Either solid fuel use | 131/456 (28.7) | Ref | 1.35 (0.95 - 1.94)^a^ | 1.03 (0.71 - 1.50) |
| Both solid fuel use | 349/1,065 (32.8) | Ref | 1.39 (0.99 - 1.96) | 1.28 (0.90 - 1.81) |

Model 3 was adjusted for age, sex, education levels, marital status, working status, household income, residence, smoking status, drinking status, and BMI.

^a^ Odds ratio (95% confidence interval) (all such value).

Table S7. Sensitivity analysis for associations of the joint of solid fuel use for cooking and heating with the progression of multimorbidity during follow-up defining electric or solar as clean fuel with the fully adjusted model (N = 5,437) (China Health and Retirement Longitudinal Study, China, 2011 - 2018).

|  | **Case of multimorbidity progression/Number (%)** | **Stable condition progression** | **Single condition progression** | **Multimorbidity progression** |
| --- | --- | --- | --- | --- |
| **Cooking and heating** |  |  |  |  |
| Both clean fuel use | 153/314 (48.7) | Ref | Ref | Ref |
| Either solid fuel use | 655/1,223 (53.6) | Ref | 1.12 (0.74 - 1.72)^a^ | 1.20 (0.92 - 1.57) |
| Both solid fuel use | 2,203/3,900 (56.5) | Ref | 1.29 (0.87 - 1.92) | 1.41 (1.09 - 1.82) |

Model 3 was adjusted for age, sex, education levels, marital status, working status, household income, residence, smoking status, drinking status, and BMI.

^a^ Odds ratio (95% confidence interval) (all such value).

Table S8. Sensitivity analysis for associations between the duration of solid fuel use for cooking from 2011 to 2018 and multimorbidity progression with the fully adjusted model (N = 5,437) (China Health and Retirement Longitudinal Study, China, 2011 - 2018).

|  | **Case of multimorbidity progression/number (%)** | **Stable condition progression** | **Single condition progression** | **Multimorbidity progression** |
| --- | --- | --- | --- | --- |
| **Duration of solid fuel use for cooking** | |  |  |  |
| 0 years | 683/1,315 (51.9) | Ref | Ref | Ref |
| 1 - 6 years | 1,444/2,614 (55.2) | Ref | 1.20 (0.95 - 1.53)^a^ | 1.19 (1.02 - 1.39) |
| 7 years or more | 884/1,508 (58.6) | Ref | 1.03 (0.78 - 1.37) | 1.36 (1.13 - 1.62) |

Model 3 was adjusted for age, sex, education levels, marital status, working status, household income, residence, smoking status, drinking status, and BMI.

^a^ Odds ratio (95% confidence interval) (all such value).
